# Gastrointestinal Stromal Tumor: Chondro-Myxoid Variant Mimicking Chondrosarcoma

**DOI:** 10.1080/13577140500043708

**Published:** 2005

**Authors:** Ashwani Rajput, Charles Levea, Marilyn Intengan, John Kane, John F. Gibbs

**Affiliations:** 1 Department of Surgical Oncology Roswell Park Cancer Institute The State University of New York at Buffalo Elm and Carlton Streets Buffalo Buffalo NY 14263 USA; 2 Department of Pathology Roswell Park Cancer Institute The State University of New York at Buffalo Buffalo NY USA; 3 Department of Surgical Oncology Roswell Park Cancer Institute The State University of New York at Buffalo Buffalo NY USA


*Sarcoma*, March/June 2005; 9(1/2): 25–28

Taylor & Francis

Taylor & Francis Group

**CASE REPORT**

### Gastrointestinal Stromal Tumor: Chondro-myxoid variant mimicking chondrosarcoma

ASHWANI RAJPUT[1], CHARLES LEVEA[2], MARILYN INTENGAN[2], JOHN KANE[1], & JOHN F. GIBBS[1]

*Departments of [1]Surgical Oncology and [2]Pathology, Roswell Park Cancer Institute and The State University of New York at Buffalo, Buffalo, NY*

SW, an 84-year-old African American female, presented to an outside institution complaining of dull, intermittent abdominal pain in the anterolateral and suprapubic regions. Decreased appetite, nausea, and general weakness accompanied this pain. Physical exam revealed a soft, non-tender mass in the left upper quadrant that extended 20 cm below the costal margin. A separate 2 cm mass, in the periumbilical area, was also palpable. A CT scan (Figure 1) was performed and revealed a large, soft tissue mass in the left upper quadrant extending from the gastroesophageal junction to the mid-abdomen. The mass seemed to originate from the stomach compressing the stomach medially, and the pancreas and spleen inferiorly and laterally. The periumbilical mass was subcutaneous and separate from the larger mass. A fine needle aspiration and biopsy of the umbilical mass was diagnosed as a myxoid chondrosarcoma. No further therapy was given at that time.

Approximately four months later, the patient presented to our institution with nausea, abdominal pain, and increased regurgitation of food. Given the clinical presentation, radiologic findings, and biopsy results, the patient was felt to have a primary sarcoma of the stomach. Since the patient was an appropriate surgical candidate, she was taken to the operating room. Upon laparotomy, a large mass, originating from a pedicle attached to the greater curvature of the stomach, was observed. The tumor was adherent to the diaphragm and to the lateral segment of the liver. No invasion of any other adjacent structures was observed. The periumbilical mass appeared to be a portion of the original tumor that had become incarcerated in an umbilical hernia defect that had fractured away from the dominant lesion.

On gross pathologic examination, a 7012 gram, multinodular, cerebreform mass, was observed to be arising from within the wall of the greater curvature of the stomach. This mass extended from near the gastroesophageal junction to the pyloris. The tumor showed expansive intra-abdominal growth and involved the omentum and anterior abdominal wall. Sectioning showed a heterogeneous, lobulated, gray-white mass with areas of necrosis, hemorrhage, and cystic degeneration (Figure 2).

The tumor had a variable histologic appearance. Portions of the tumor were relatively hypocellular with a vaguely nodular, but prominent chondroid matrix. Dispersed within this matrix were discohesive epithelioid cells. Focal areas of myxoid cystic degeneration were also present. The majority of the tumor was extremely cellular containing sheets of epithelioid cells within a chondroid matrix. Additionally, other areas had more spindle-shaped cells. These cells showed nuclear palisading and were aligned in a perivascular distribution. Focally, these cells formed microcysts. Tumor cell necrosis, hemorrhage, and high mitotic activity (65/50 hpf) was observed in these spindle cell areas. The differential diagnosis of this tumor upon H&E staining included a chondrosarcoma and a gastrointestinal stromal tumor (Figure 3). S100 staining, which should be positive in >95% of chondrosarcomas, was negative. The tumor cells were positive for c-kit (CD-117), CD-34, CD-99 (mic2), and smooth muscle actin (SMA) (Figure 3). This staining pattern confirmed

Correspondence: Ashwani Rajput, MD, GI Surgical Oncology, Roswell Park Cancer Institute, Elm and Carlton Streets Buffalo, NY 14263. Tel: 716-845-3565. Fax: 716-845-1278. E-mail: ashwani.rajput@roswellpark.org

ISSN 1357–714X print/ISSN 1369–1643 online © 2005 Taylor & Francis Group Ltd
DOI: 10.1080/13577140500043708

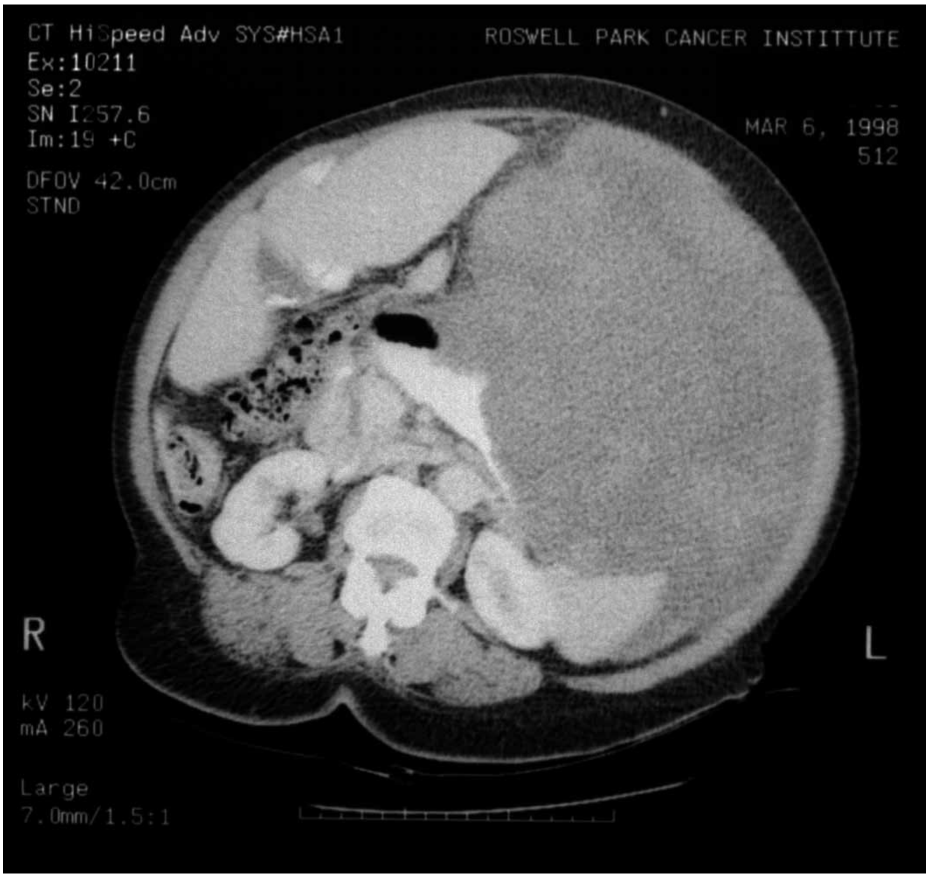

Figure 1. Axial CT scan image showing the obvious large, left upper quadrant mass.

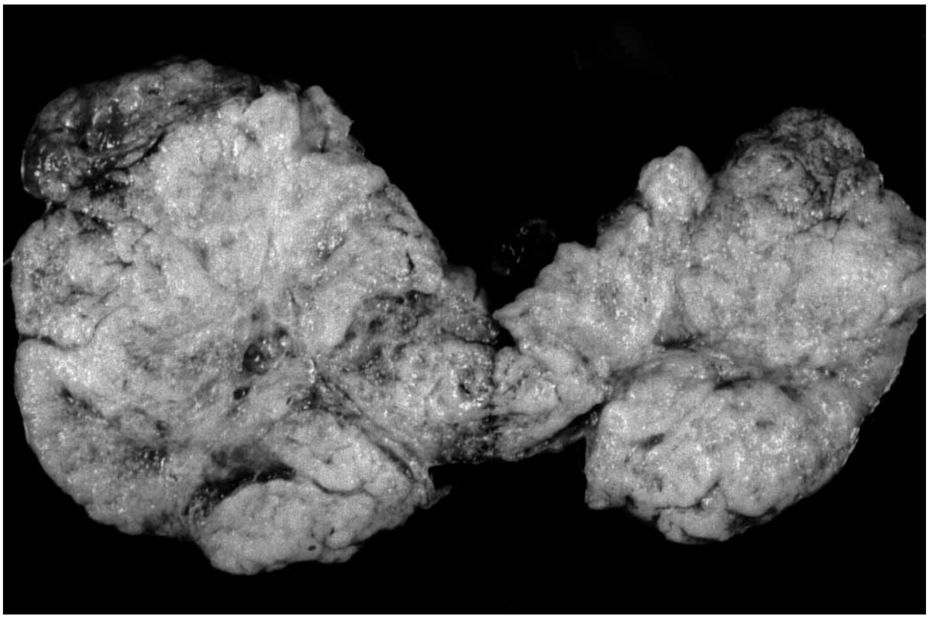

Figure 2. Gross pathologic specimen showing heterogeneous, lobulated mass with areas of necrosis, hemorrhage, and cystic degeneration.

the diagnosis of a gastrointestinal stromal tumor (GIST).

### Discussion

We describe an intraabdominal gastrointestinal stromal tumor (GIST) arising from the stomach with chondro-myxoid features, which has not been previously reported. Its histologic appearance was originally felt to represent a chondrosarcoma, which does not arise from the gastric wall. We originally encountered this lesion prior to the utilization of CD99 and CD117 immunohistochemical staining for GIST tumors. Thus, the emergence of the

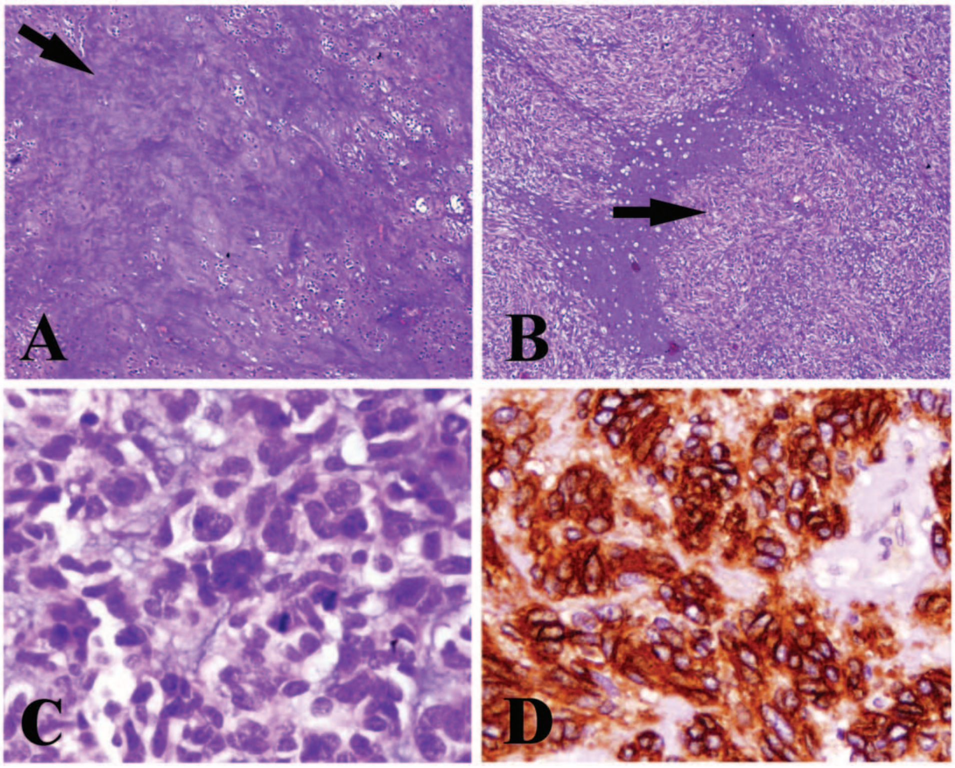

Figure 3. Hematoxylin and Eosin stain showing (a) chondroid areas; (b) low power view epithelioid cells demonstrating more cellular areas and chondroid areas; (c) high power of mitoses in the spindle cell areas; (d) immunohistochemical stain showing c-kit.

immunostains allows us to identify of a chondroid variant of GIST.

GIST was once a term applied to a diverse group of neoplasms. Historically, this term was used to describe all mesenchymal tumors of the gastrointestinal (GI) tract. Since this term was applied to a group of tumors instead of just one, there was a considerable debate in regards to this ‘‘single tumor's'' histogenesis. It is now clear that the term GIST refers only to those tumors that arise from the interstitial cells of Cajal or their precursors, the gastrointestinal pacemaker cells [1–3].

Contributing to this historical confusion over the histogenesis of GIST is their variable light microscopic appearance. The majority of these tumors are composed of either epithelioid or spindle cells. A combination of epithelioid and spindle cells can be present in the same tumor; however, one of these morphologies usually predominates. The epithelioid cells have a solid, sheet-like architecture and may show focal vacuolar change mimicking signet ring cells. They also may contain peripherally located hyperchromatic nuclei. The spindle cells may have a fibrillary or myxoid stroma, form microcysts, become arranged in fascicles, show nuclear palisading, show perivascular hyalinization, or contain skenoid fibers within their stroma.

The immunohistochemical staining pattern of GIST varies with their location within the gastrointestinal tract. Miettinen et al. [4], analyzed 92 c-kit positive (CD117) GIST from the stomach. Of these tumors, 90% were CD-34 positive, 30% were smooth muscle actin positive, 3% were desmin positive, and 2% were S100 positive. These results confirm the ultrastructural finding that the majority of GIST exhibit a primitive myoid phenotype. Recently, CD-99 has been shown to be positive in 89% (24 of 27) of GIST [5]. Therefore, a GIST will typically be c-kit, CD-34, and CD-99 positive, and both desmin and S100 negative.

The stroma of GIST may contain hemorrhage, hyalinization, and myxoid changes. Suster [6] examined 9 cases of GIST with prominent myxoid stromal backgrounds. These tumors lacked both the immunohistochemical and ultrastructural features of neural or ganglionic differentiation. They exhibited a primitive mesenchymal phenotype with features of immature smooth muscle cells. The myxoid changes seen in these lesions may represent a secondary non-specific reaction of the stroma to the tumor cells, may be a degenerative change, or may be directly produced by the tumor cells themselves. Although the lesion described in this report also has an extensive myxoid stroma and a myoid phenotype, it differs from the lesions that Suster described as it also has extensive areas of a chondroid stromal matrix. Therefore, this tumor is a distinctive morphologic variant of GIST that is important for physicians to recognize.

Gain-of-function mutations in exon 11 of the c-kit proto-oncogene and c-kit protein overexpression are found in GIST [7, 8]. The c-kit gene encodes for a receptor for a growth factor called stem cell factor. This receptor is a tyrosine kinase that regulates cell growth and survival. Gain-of-function mutations of the c-kit gene result in ligand-independent constitutive activation of the tyrosine kinase, which plays a role in tumor promotion.

The metastatic potential of GIST is influenced by tumor location as well as by pathologic criteria. Gastric tumors, by virtue of their location, have the best prognosis. The tumors are best divided into groups with differing risks for aggressive behavior. Morphologic criteria for high risk for aggressive behavior include lesions greater than 5 cm with mitotic rates of greater than 5/50 high power fields or any tumor greater than 10 cm or any size with a mitotic rate greater than 10/50 high power fields [9]. Stromal tumors with a size less than 2 cm and less than 5 mitoses per 50 high power fields have a very low risk for aggressive behavior, and tumors with a size less than 5 cm and a mitotic rate of 6–10/50 high power fields or a size between 5 and 10 cm and a mitotic rate of less than 5 per 50 high power fields are considered intermediate risk for aggressive behavior.

It should be noted that a GIST, although having a high risk for aggressive behavior, may behave indolently. On the other hand, a tumor diagnosed as a GIST with low risk for aggressive behavior may recur and metastasize. It still has a risk, albeit low, of metastasizing.

Primary gastrointestinal mesenchymal tumors that differentiate along myoid, neural, and ganglionic lines have been shown to have variable biologic behavior. For example, in the case of a GIST with a myxoid matrix, the differential diagnosis includes schwannoma, which behaves indolently, and gastrointestinal autonomic nerve tumors, which are known for their aggressive clinical course. GIST with a myxoid matrix may behave either indolently or aggressively (as outlined above). It is important to differentiate among these tumors since GIST can be treated effectively with imatinib mesylate (STI571), an inhibitor of the c-kit receptor tyrosine kinase [10–13].

### Conclusion

This case illustrates a GIST that shows prominent chondro-myxoid differentiation. This was an indolent tumor amenable to surgical resection. This unique morphological variant of GIST has not been previously reported in the literature. The prognosis and treatment of GIST differs from myxoid chondrosarcomas, and, thus, it is important to differentiate between the two tumors.

### References

1. Somerhausen NDSA, Fletcher CDM. Gastrointestinal stromal tumours: an update. Sarcoma 1998;2:133–141.
2. Miettinen M, Lasota J. Gastrointestinal stromal tumors (GISTs): definition, occurrence, pathology, differential diagnosis and molecular genetics. Pol J Pathol 2003;54(1):3–24.
3. Pidhorecky I, Cheney RT, Kraybill WG, Gibbs JF. Gastrointestinal stromal tumors: current diagnosis, biologic behavior, and management. Ann Surg Oncol 2000;7(9): 705–712.
4. Miettinen M, Sobin LH, Sarlomo-Rikala M. Immunohistochemical spectrum of GISTs at different sites and their differential diagnosis with a reference to CD117 (KIT). Mod Pathol 2000;13(10):1134–42.
5. Shidham VB, Chivukula M, Gupta D, Rao RN, Komorowski R. Immunohistochemical comparison of gastrointestinal stromal tumor and solitary fibrous tumor. Arch Pathol Lab Med 2002;126(10):1189–1192.
6. Suster S, Sorace D, Moran CA. Gastrointestinal stromal tumors with prominent myxoid matrix. Clinicopathologic, immunohistochemical and ultrastructural study of nine cases of a distinctive morphologic variant of myogenic stromal tumor. Am J Surg Pathol 1995;19(1):59–70.
7. Hirota S, Isozaki K, Moriyama Y, Hashimoto K, Nishida T, Ishiguro S, Kawano K, Hanada M, Kurata A, Takeda M, Tunio GM, Matsuzawa Y, Kanakura Y, Shinomura Y, Kitamura Y. Gain-of-function mutations of c-kit in human gastrointestinal stromal tumors. Science 1998;279:577–580.
8. Nakahara M, Isozaki K, Hirota S, Miyagawa J-I, Hase-Sawada N, Taniguchi M, Nishida T, Kanayama S, Kitamura Y, Shinomura Y, Matsuzawa Y. A novel gain-of-function mutation of c-kit gene in gastrointestinal stromal tumors. Gastroenterology 1998;115:1090–1095.
9. Fletcher CDM, Berman JJ, Corless C, Gorstein F, Lasota J, Longley BJ, Miettinen M, O'Leary TJ, Remotti H, Rubin BP, Shmookler B, Sobin LH, Weiss SW. Diagnosis of gastrointestinal stromal tumors: A consensus approach. Human Pathol 2002;33(5):459–465.
10. Joensuu H, Roberts PJ, Sarlomo-Rikala M, Andersson LC, Tervahartiala P, Tuveson D, Silberman SL, Capdeville R, Dimitrijevic S, Druker B, Demetri GD. Effect of the tyrosine kinase inhibitor STI571 in a patient with a metastatic gastrointestinal stromal tumor. N Engl J Med 2001;344(14): 1052–1056.
11. Rajput A, Kraybill WG. Clinical trials and soft tissue sarcomas. Surg Oncol Clin N Am 2003;12(2):485–497.
12. Eisenberg BL. Imatinib mesylate: A molecularly targeted therapy for gastrointestinal stromal tumors. Oncology 2003;17(11):1615–1620, discussion 1620, 1623, 1626 passim.
13. Duffaud F, Blay JY. Gastrointestinal stromal tumors: biology and treatment. Oncology 2003;65(3):187–197.

